# Health systems strengthening in German development cooperation: making the case for a comprehensive strategy

**DOI:** 10.1186/s12992-016-0215-3

**Published:** 2016-12-03

**Authors:** Khullat Munir, Ilse Worm

**Affiliations:** German Institute for Development Evaluation (DEval), Bonn, Germany

**Keywords:** Health systems strengthening, Health systems, Health policy, Accountability, Effectiveness, Germany, International cooperation, Foreign aid

## Abstract

In the aftermath of the Ebola crisis and with renewed attention to resilient health systems, the process of improving approaches of global health actors to health systems strengthening is of great relevance. Despite the increased amount of attention paid to health systems strengthening, there is no standard definition of this concept among global health actors. Germany is no exception. Though there have been recent commitments to increase resources allocated to health systems strengthening, German Development Cooperation has no comprehensive strategy for its pursuit. This article sheds light on how HSS can be more clearly defined in German bilateral health cooperation, and makes a case for the adoption of a comprehensive HSS strategy.

A strategic analysis of the German Development Cooperation’s orientation towards health systems strengthening reveals the focal areas and cross-cutting approaches of Germany’s engagement with the health systems strengthening. These elements are then linked to the building blocks of the health system, as defined by the World Health Organization. The resulting framework should be further elaborated with data from implementation to develop a comprehensive health systems strengthening strategy. Both the U.S. and U.K. have also recently reviewed their own health systems strengthening efforts and concluded with clear statements on the need for well-defined health systems strengthening strategies. We argue that in developing such strategies, a sound base can be provided by a health systems strengthening framework that is based on both strategic inputs from existing health policies as well as implementation experiences.

Despite its limitations, the current framework provided in this review shows how the German Development Cooperation intends to enact health systems strengthening, and can thus have several uses: (1) ensure the systemic nature of health systems strengthening planning and implementation (2) guide the consistency of Germany’s approach to health systems strengthening within partner countries and (3) create theories of change for health evaluations. This approach can potentially be applied to systematically interpret health systems strengthening strategies of other global health actors, leading to better coherence and accountability in health development efforts.

## Background

In the past decade the concept of health systems strengthening (HSS) has pervaded the international development sphere. With the launch of the World Health Organization (WHO) publication *Everyone’s Business: Strengthening Health Systems to Improve Health Outcomes* in 2007, the health systems building blocks framework was released and aimed to broaden the focus of global health from disease control programs to health systems [1]. Since then, a number of bi- and multilateral organizations have placed increasing efforts in the strengthening of health systems. Momentum for HSS has increased due to recent pandemics and natural disasters. HSS has remained at the front and centre of the global health agenda. It was one of the health priorities of the 2015 G7 Summit [2], and is a necessity in the achievement of universal health coverage, a focus of Sustainable Development Goal (SDG) 3. With vast resources flowing into HSS worldwide, there is increased need for accountability, efficiency and effectiveness. However, fulfilling this need is problematic due to the lack of unity among global health actors on a standard definition and means of assessing HSS.

HSS constitutes an important aspect of the German Development Cooperation’s (GDC) multilateral and bilateral engagement in health development. Though HSS is a priority of the health sector strategy of the German Federal Ministry for Economic Cooperation and Development (BMZ), GDC has of yet no comprehensive strategy for the pursuit of HSS.

This article aims to shed light on how HSS can be more clearly defined in German bilateral health cooperation. It embodies two objectives: first, to unpack the concept of HSS in current German Development Cooperation policy and identify its core elements. Second, to elaborate an analytical framework that can be used to develop a comprehensive strategy for HSS. Though this analysis produces results specific to the GDC, the analytic approach utilized would potentially be of use for other global health actors seeking to further sharpen their approach to HSS.

Our analysis of the strategic orientation of HSS within the GDC builds on a body of work that aims to categorically understand HSS [3–7] and gives insight on how HSS can be structurally defined. Undertaking these efforts enables greater tangibility to the concept of HSS, making it less of a “container concept” that has often left health development efforts weakened [8].

The first section of the article outlines the methods undertaken in the analysis. The following sections present the findings of our analysis. To set the stage, GDC´s contributions to global health are presented with a focus on bilateral official development assistance (ODA) funded by the BMZ. The third and fourth sections analyse GDC´s strategic orientation towards HSS as reflected in BMZ policies and strategies. The fifth section shows how this orientation fits in the WHO health systems building blocks framework. The sixth section briefly sheds light on other countries’ approaches in reviewing their HSS efforts and investments. In conclusion we discuss how the framework applied in our analysis could be used and further elaborated to develop a comprehensive HSS strategy.

## Methods

This review is based on two recently published papers by the German Institute for Development Evaluation (DEval), the first of which is a portfolio analysis of GDC contribution to global health in 2002–2013. The main source of data used in the analysis was the Creditor Reporting System (CRS) Aid Activity Database of the OECD/DAC, which is the most comprehensive dataset available for ODA.

**Fig. 1:**
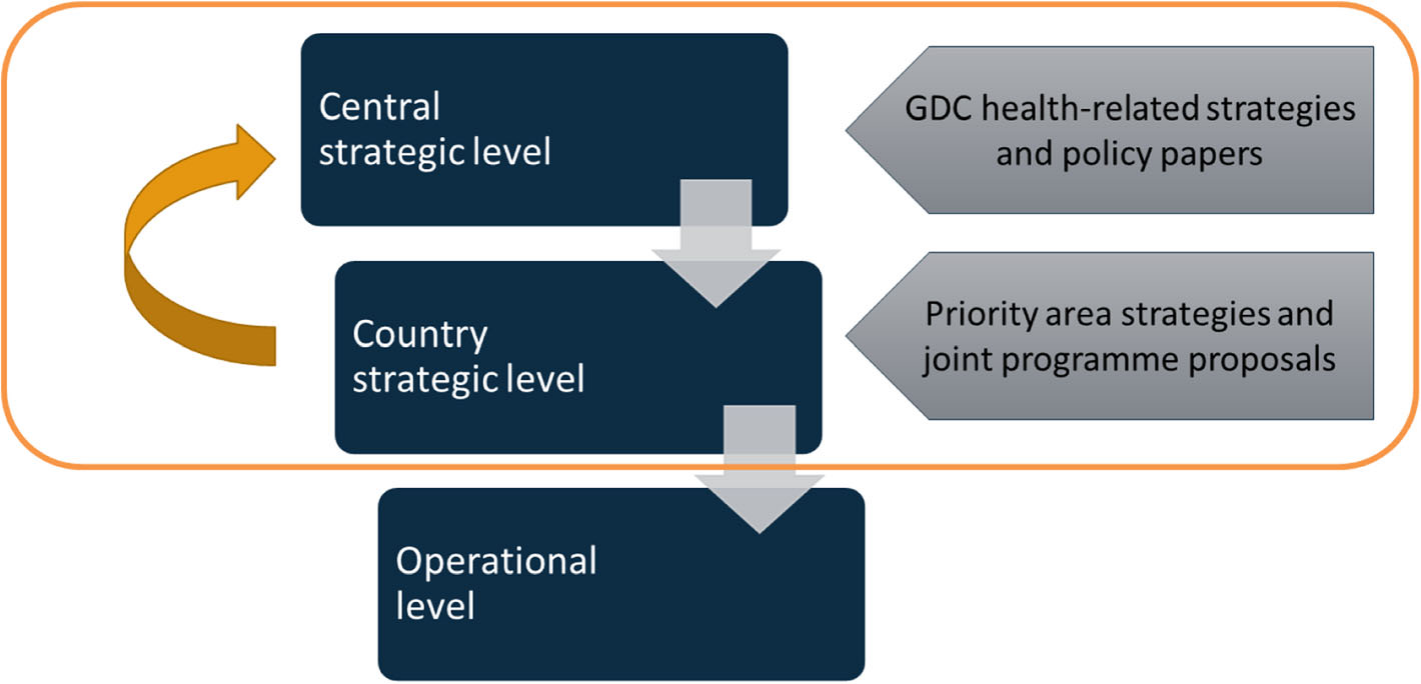
Analytical approach. (Legend) Source: [15]

The second paper is a qualitative analysis of GDC’s strategic orientation towards HSS, as reflected in key policies and strategies. In a first step the analysis covered BMZ strategic documents at central level. Document selection entailed the incorporation of all sector strategies and policies published by the BMZ under the category of health., as well as the inter-ministerial global health concept of the German Government.^1^ This first round of analysis shows that the GDC strategically pursues HSS through three focal areas and four cross-cutting approaches. These focal areas and cross-cutting approaches were then used in a second step to guide the analysis of country strategic documents (including priority area strategies in health and joint programme proposals) in eight partner countries. These strategic documents outline the GDC cooperation strategy in the health sector of the partner countries and orient operational planning. Eight partner countries (Cambodia, Kenya, Malawi, Nepal, Pakistan, South Africa, Tanzania, Vietnam), and one region (Central Asia) were chosen on the basis of the following criteria: their partnership with the GDC entailed health as one of their priority areas of overall development cooperation, and health would continue to be a priority area for the foreseeable future. The analysis examined the extent to which focal areas and cross-cutting approaches defined at the central level were reflected within country strategic documents (Fig. 1).

After having extracted and categorized the focal areas and cross-cutting approaches relevant to HSS at the central strategic and country levels, the analysis connected them to the WHO health system building blocks framework.

## Findings

### Germany as a global health player

As the third largest bilateral donor for global health in 2014, Germany plays an influential role in the health development sector. In just over a decade, German ODA for health has tripled in absolute value, rising to approximately EUR 786 million in 2013 [9]. In the period 2002–13, multilateral ODA comprised the majority of German health ODA (55%). Germany was also the fourth largest country donor in terms of multilateral health ODA in 2013, with top recipients including The Global Fund, EU institutions, International Development Association, WHO and other UN organizations.

**Fig. 2:**
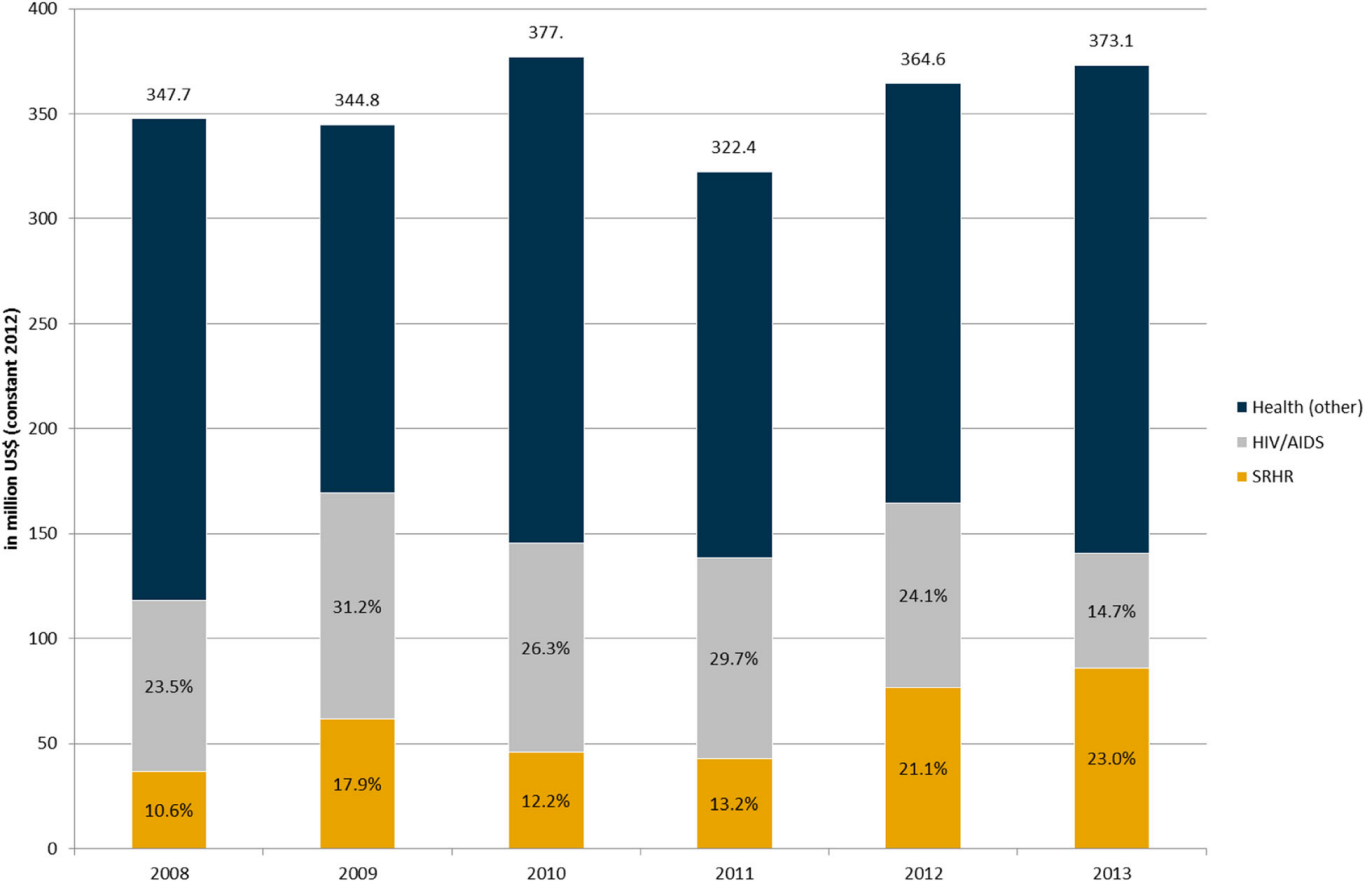
Bilateral BMZ-supported health ODA disbursements over time by thematic area, 2008–13. (Legend) Source: [9]. Note: Total health ODA is defined as sector codes 121, 122 and 130; HIV is defined as purpose code 13040; SRHR is defined as aggregate of purpose codes 13020, 13030, and 13081

The BMZ Sector Strategy for German Development Policy in the Health Sector [10] places focus on three strategic areas: HSS, HIV/AIDS and other infectious diseases, and sexual and reproductive health and rights (SRHR). Analysis of bilateral German health ODA in 2008 using CRS data reveals that HIV/AIDS and SRHR accounted for 24.7% and 16.4%, respectively.^2^ The remaining portion, classified as “Health (other)”, accounted for 58.9% (Fig. 2).

Limitations of the CRS database prevent valid analysis and tracking of HSS interventions through health ODA. Each aid activity within the CRS database is allocated a single purpose code, which is usually either very broad or highly intervention-specific. In the case of aid activities in the health sector, this is a very limiting means of categorization. HSS components may be present in any given project/ programme, ultimately resulting in the analysis of HSS funding to become increasingly complex. For instance, a project focused on service delivery for HIV/AIDS patients may also place emphasis on the institutional capacity development of health workers that strengthens the health system as a whole. Similarly, support for a midwife training program might also aim for the improvement of the overall health referral system. With such cases, tracking the HSS components of a donor’s entire inventory of aid activities is a complex task. As a result, there is currently no means of measuring HSS flows and holding GDC accountable for its efforts in supporting HSS.

Despite lacking accountability mechanisms for HSS, donors are allocating greater resources towards it. Chancellor Angela Merkel announced a 200 million EUR commitment for HSS at the 2015 World Health Summit [11], which was followed up with the launch of the Healthy Systems – Healthy Lives Initiative in September 2015. This initiative aims to facilitate the development of a comprehensive understanding of HSS among various stakeholders, such as multilateral and bilateral partners, governments, civil society, private sector and academia, and to improve the coordination and effective support to HSS by the international community. To this end a consultative process among global health actors to elaborate a roadmap for HSS is currently taking place [12].

### GDC´s strategic orientation towards HSS

Overall, the GDC health-related strategies and policy papers at the central level acknowledge HSS as a precondition for universal health coverage as well as for the achievement of the health-related MDGs. By contributing to the realization of the right to health, HSS is seen as a means of achieving sustainable and inclusive health outcomes [10].

Although HSS is one of the three priority areas of the BMZ Sector Strategy for German Development Policy in the Health Sector [10], in contrast to HIV [13] and SRHR [14] there is up to now no strategic concept of how GDC intends to pursue HSS. However, HSS is mentioned in most policy and strategy papers at the central strategic level [15]. It is mainly defined through the support GDC provides to three focal areas:

- Human resource development for health
- Solidarity based health financing and social health protection
- Institutional and organisational development of national health systems

These focal areas are supplemented by four cross-cutting approaches:k

- Private sector cooperation
- Vertical programme integration
- Inter-sectoral cooperation
- Human rights based approach

For each focal area and cross-cutting approach the strategies include a range of specific measures that potentially contribute to HSS, although these are not always labelled explicitly as HSS (Table 1).

**Table 1:** Focal areas and cross-cutting approaches in GDC approach to HSS

Focal areas and cross-cutting approaches	Examples of specific measures in GDC Strategies
Human resource development for health	- Provision of training and professional development - Policy dialogue at national and international levels on migration policies and recruitment of health workers, including the promotion of the WHO Code of Practice on International Recruitment of Health Personnel - Support to and cooperation with the Global Health Workforce Alliance - Support establishment of legal frameworks and acceptable working conditions for health professionals
Solidarity based health financing and social health protection	- Support for health finance system reform in partner countries - Development of various health financing models, including taxation based systems, social health insurance schemes, conditional cash transfers, demand subsidization, etc. - Support to and cooperation with the Providing for Health Initiative
Institutional and organizational development of national health systems	- Support decentralization of health systems - Support development and implementation of health sector reforms - Promote participation of target group and community in planning and M&E of health services - Support harmonization and sector-wide approaches in the health sector - Support to and participation in the International Health Partnership (IHP+) at global and country levels - Support expansion and rehabilitation of infrastructure at the primary and secondary level
Private sector cooperation	- Support development of public-private partnerships (with private actors both in Germany and in partner countries), especially for the subsidization of health services and medical products - Development and strengthening of private production, supply and distribution channels to provide medical products and drugs - Support to and participation in the Harnessing Non-State Actors for Better Health for the Poor Initiative
Vertical programme integration	- At service delivery level: promote integration of SRH and HIV prevention; promote linkages between internal medicine and HIV services - At national level: support development of comprehensive strategies that integrate HIV activities with those of Tuberculosis or SRHR; support coordination of bilateral and multilateral donor contributions for vertical programmes into national systems - At international and country level: work through the Global Fund and other global health initiatives that incorporate HSS in their work; provision of technical assistance for Global Fund applicants and recipients to optimize funds
Inter-sectoral cooperation	- Focus on prevention and health promotion - National and international levels: support the development of “health in all policies” (i.e. integration of health aspects into trade or migration policies); promote policy coherence and development of laws and regulations to address risk factors for non-communicable diseases (i.e. malnutrition, tobacco consumption)
Human rights-based approach	- Support development and implementation of inclusive health strategies that take into account the needs of marginalized and vulnerable groups - Advocacy, information, education and communication to overcome stigma and discriminatory practices - Development of mechanisms to strengthen patient rights, complaint and redress mechanisms - Support to participatory planning and decision-taking processes at all levels

Source: [15]

### Strategic orientation of GDC towards HSS within partner countries

Overall, the strategic orientation of GDC towards HSS within partner countries is largely in line with the GDC health policies and strategies at the central level. However, it differs in the extent to which certain focal areas and cross cutting approaches for HSS are addressed, mirroring the variance in country-specific context and negotiation processes between partner countries and GDC.

Although not all analysed country partnerships in health take HSS into explicit consideration, the majority touch on various building blocks of health systems and their respective strengthening. Social health protection and institutional and organizational development of national health systems are most frequently addressed, followed by human resource development and quality management. Quality management is a focal area in several country strategic documents and is related explicitly to HSS, yet is not addressed as a constitutive element of HSS within the GDC central health strategies. Consequently, the results of this analysis make a clear case for the inclusion of quality management within a comprehensive HSS strategy in the GDC.

Of all the focal areas and cross-cutting approaches analysed for, private sector cooperation is most often mentioned explicitly in the context of HSS. Though specific interventions for private sector cooperation are not always detailed, usual interventions listed include strengthening of regulatory frameworks for the engagement of private health care providers and support to public-private partnerships. Vertical programme integration is only mentioned and related to HSS in the Sub-Saharan context, where integration of HIV and SRHR in other focal areas, such as quality management, is a constitutive element of a few joint health programmes.

Inter-sectoral cooperation plays a very marginal role at the country strategic level. While inter-sectoral cooperation is clearly identified by the GDC strategic documents at the central level as an essential component of HSS, the lack of priority given to it in the country strategic documents shows a potential missed opportunity.

### GDC’s strategic orientation towards HSS in light of the WHO framework

**Fig. 3:**
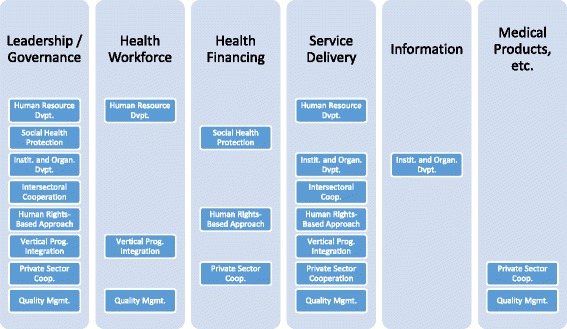
GDC pursuit of HSS categorized by health system building blocks. (Legend) Source: [15]

Both at central and country level, the GDC approach towards HSS embraces the WHO health systems building blocks framework. Figure 2 reflects the main links between the focal areas and cross-cutting approaches and the WHO health system building blocks, as entailed in the GDC strategic documents. As such, it is built solely off of the policy analysis presented here and does not incorporate data from *implementation* of health interventions (Fig. 3).

The framework in Fig. 3 suggests that the building blocks leadership/governance, health workforce, health financing and service delivery are seemingly the most important for the GDC approach to HSS, with the remaining building blocks playing a relatively less significant role.

Furthermore, this building blocks categorisation suggests that the private sector cooperation and quality management deal most comprehensively with the health system. However, this picture remains incomplete without incorporating the reality of programme implementation. The multiple links between the health system buildings blocks, the interaction with the broader environment and the overall dynamic of change still need to be conceptualized in a guiding strategic framework. Important issues, such as the extent to which these approaches contribute to achieve equitable health outcomes, thus remain elusive.

### HSS reviews by other global health actors

In the aftermath of the Ebola epidemic other bilateral global health actors have given renewed attention to HSS as well. In 2014, the U.S. and the U.K. reviewed their own approaches to HSS with the aim to improve resource allocation, and produce recommendations for improving efficiency and effectiveness of HSS efforts within their development policies and engagement with partner countries [16, 17]. Similar to the findings of this analysis, both reviews found difficulty in tracking resources for HSS.

The U.K. review of Department for International Development (DFID) policy and HSS efforts concluded that despite the prominence of its global health work, there is need for a global strategy that would guide HSS efforts in partner countries. Such a strategy would also be necessary to guide DFID-supported multilateral organizations in ensuring their efforts go toward the strengthening of health systems at a strategic level. Similar to the recommendations of this paper, the review encourages the elaboration of a strategy that defines how DFID intends to strengthen health systems through its work [16]. In reaction, the U.K. government committed to the formation of an HSS framework, which will define the areas of focus for work in developing countries and globally. Furthermore, the framework intends to incorporate the SDGs and measurement of progress towards UHC [18].

Taking their analysis one step further, the review of American investments in HSS resulted in concrete recommendations for a HSS strategy. As stated in the review, components of such a strategy should include (1) emphasis on technical cooperation and country ownership, with more attention paid to measurement of health outcomes, (2) greater investment in global health research and professional training for students in developing countries, and (3) the implementation of rigorous, external impact evaluations of global health projects involving technical innovation or new models for service delivery [17].

Both reviews from the U.K. and U.S. voice a common need to better understand how technical and financial cooperation can lead to the strengthening of health systems, and strongly encourage the formation of strategies to this effect. Likewise, a German HSS strategy, the need for which has been established in the DEval publications on German contributions to health, would fall well in line with the recent developments of other major bilateral global health actors.

## Conclusions

The analysis undertaken here of GDC health-related policies and strategies exemplifies a systematic way to make the broad concept of HSS tangible and more accessible. In providing a systematic way to understand and categorize HSS efforts, this analytic approach provides a potential template for streamlining and refining HSS efforts of other global health actors. As the global development arena shifts focus towards SDGs and universal health coverage, and with increasing emphasis being placed on *resilient* health systems, HSS will likely be a central development issue for the foreseeable future. In this context, it is clear that HSS efforts among global health actors must be coordinated, strategic, and effective. An initial step in this direction is obtaining a clear understanding of how each actor pursues HSS and its strengths in doing so.

As revealed in the analysis, HSS is a strong tenet of the GDC’s contribution to global health and recent commitments have been made to increase Germany´s support to HSS. However, there are up to now no means of measuring German ODA to HSS, thus making it difficult to hold Germany accountable for its HSS efforts. Furthermore, the analysed strategic documents do not convey yet a clear picture of how GDC intends to contribute to HSS. A comprehensive and unified HSS strategy would enable GDC to streamline its policy planning and resource allocation.

Despite its limitations, the framework in Fig. 3 provides a hitherto unseen reflection of the GDC’s strategic orientation towards HSS. Hence, it presents a starting point for developing a comprehensive HSS strategy and can have a number of uses:

### Ensuring the systemic nature of HSS planning and implementation

The framework shows the systemic nature of HSS, exemplifying that each focal area or cross-cutting approach supported by GDC functions through a number of building blocks. For example, without engaging policy makers to legislate improved working conditions for health workers (represented by the intersection of the focal area “human resource development” and the leadership/governance building block), further capacity development of health workers (represented by the intersection of the focal area “human resource development” and the service delivery building block) might remain limited.

Conceptualization of the framework needs to go beyond the first steps taken in our analysis of health-related policies and strategies. The GDC focal areas and cross-cutting approaches for HSS should be further linked with the health system building blocks and reflect the realities of GDC implementation. More attention should also be paid to the relationships with sectors other than health, thus defining better how the cross-cutting approach “inter-sectoral cooperation” relates to HSS.

To this end a more pronounced systems’ perspective is needed. Rather than focusing on single elements or building blocks of the health system, a systems’ perspective views them as interacting parts of a whole dynamic, and places this dynamic within the context of sectors outside of health [19].

### Guidance for the consistency of GDC’s approach to HSS within partner countries

While global health actors are increasingly making the shift towards developing frameworks for HSS, it must be acknowledged that HSS is context-specific in its implementation. As such, it cannot be applied with a “one size fits all“ template across all settings.

However, by utilizing the broad view of HSS orientation in this framework, a more unified approach can be taken when negotiating and planning engagement in the health sector of partner countries. A transparent framework would benefit partner countries by allowing them a broad but succinct perspective of the GDC’s strategic orientation towards HSS, equipping them with more resources to enter into negotiations for GDC engagement in the health sectors of their country. It would also enable GDC to position itself better among other development partners.

### Basis for theories of change to be used in future health evaluations

Given the fact that this framework is built off of the GDC health-related strategies and policies, it is a reflection of what the GDC aims to address through each of its approaches to HSS. Elaboration of this framework within an HSS strategy would enable the GDC to define more clearly how it contributes to HSS and what the intended multiple effects of HSS are. As such, the framework could be useful in the development of theories of change for future health evaluations.

Overall, the lack of defined focus on HSS within the GDC strategies calls for change. This is especially relevant in light of the fact that Germany’s newest health commitment and global health initiative are specifically focused on HSS. The fulfilment and effectiveness of this commitment, as well as the continued political priority given to HSS, cannot be truly measured without the guidance of a unified and comprehensive HSS strategy.

## Abbreviations

BMZ: German Federal Ministry for economic cooperation and development
CRS: Creditor reporting system
DEval: German Institute for development evaluation
DFID: Department for international development
GDC: German development cooperation
HSS: Health systems strengthening
ODA: Official development assistance
OECD/DAC: Organisation for economic cooperation and development/ development assistance committee
SDG: Sustainable development goal
SRHR: Sexual and reproductive health and rights
WHO: World Health Organization

## Footnotes

1 A full list of the strategies included in this review are found in the annex of the DEval publication “Health Systems Strengthening in German Development Cooperation” [16].

2 Only German ODA funded by the BMZ was incorporated in this analysis. In the period 2008-2013, the BMZ contributed to an average of 84% of total German health ODA.
